# Supplementary material for: Health economic evaluation of noninvasive prenatal testing and serum screening for down syndrome
Source: PLoS One. 2022 Apr 14;17(4):e0266718. doi: 10.1371/journal.pone.0266718 (PMC9009700; doi:10.1371/journal.pone.0266718)
Supplement: Attachment — Submitted filename: Response to Reviewers.docx [file pone.0266718.s002.docx]

Dear Editors and Reviewers:
Thank you for your letter and comments on our manuscript entitled “Health Economic Evaluation of Noninvasive Prenatal Testing and Serum Screening for Down Syndrome”. (ID: PONE-D-21-11149).
Those comments are all valuable and very helpful for revising and improving our paper, as well as the important guiding significance for our researches. We have studied comments carefully and have made correction which we hope to meet with approval. Revised portion are marked in red in the paper. The main corrections in the paper and the responds to the reviewer’s comments areas flowing.

We would like to express our great appreciation to you and reviewers forcomments on our paper. Looking forward to hearing from you.
Thank you and best regards.
Yours sincerely,
Gefei Xiao
E-mail: xgf8111_cn@hotmail.com

**Response:**

1- the first mentioning of uE3 is now moved to line 81 (this part was not included in your original submission), hence you should the full name of this abbreviation accordingly.

Response：the full name of hormone uE3 is “*unconjugated estriol*”. It has been added in line 83.

2- Your response to my 3rd comment is inadequate : (It has been mentioned in line 206.“For the 10 cases missed in first-trimester screening, calculation without NT and with the serum indicators PAPP-A and F β-hCG identified 4 cases, and the detection rate was increased to 62.5% (10/16) (Table 1).”). This doesn't necessarily imply significance. There has to be a p-value of at least <0.05 based on a suitable statistical test to conclude significant difference.

# Response：We added statistical test result in line 205, and analyzed the reason in line 262 of discussion.

3- Have the authors made all data underlying the findings in their manuscript fully available?

Response：We have added “Availability of data and materials” in line 340.
