# Supplementary material for: Health economic evaluation of noninvasive prenatal testing and serum screening for down syndrome
Source: PLoS One. 2022 Apr 14;17(4):e0266718. doi: 10.1371/journal.pone.0266718 (PMC9009700; doi:10.1371/journal.pone.0266718)
Supplement: Attachment — Submitted filename: Response to Reviewers.docx [file pone.0266718.s003.docx]

Dear Editors and Reviewers:
Thank you for your letter and comments on our manuscript entitled “Health Economic Evaluation of Noninvasive Prenatal Testing and Serum Screening for Down Syndrome”. (ID: PONE-D-21-11149).
Those comments are all valuable and very helpful for revising and improving our paper, as well as the important guiding significance for our researches. We have studied comments carefully and have made correction which we hope to meet with approval. Revised portion are marked in red in the paper. The main corrections in the paper and the responds to the reviewer’s comments areas flowing.

We would like to express our great appreciation to you and reviewers for comments on our paper. Looking forward to hearing from you.
Thank you and best regards.
Yours sincerely,
Gefei Xiao
E-mail: xgf8111_cn@hotmail.com

**Response:**

1- This is a topic where there are ethical issues involved, concerning the rights of those living with disabilities. I request the authors to refer briefly to this literature in the Introduction (a couple of sentences plus references would suffice).

# Response：Because of religious beliefs, legal regulations, medical payment and other factors, different regions adopt different strategies when choosing state-sponsored DS screening. In China, termination of pregnancy is legally permitted when the fetus is diagnosed to be disabled before birth. It has been description in line 278.

2- It would be helpful in the Discussion section to note that the results for China may not transfer readily to other countries. In some other countries, late detection of Down's syndrome may be too late for termination of the pregnancy to be legally permitted. Even where it is legally permitted, not all parents may exercise the choice to terminate the pregnancy. I don't know if this is an option in China (would Zhuhai Municipality permit the parents to continue on with the pregnancy?). In these circumstances the parents still benefit from screening in being able to prepare for the birth of a differently-abled child, but it would affect the economic costs and benefits of the outcome. Again, a couple of sentences qualifying the findings would suffice.

# Response：If the fetus is diagnosed with DS, before the fetus is born, the pregnant woman has the right to decide whether to terminate the pregnancy or give birth to the fetus.We have added your comments to lines 283, 324 and 339.
